# Facilitating the Informed Consent Process Using Teleconsent: Protocol for a Feasibility and Efficacy Study

**DOI:** 10.2196/11239

**Published:** 2018-10-17

**Authors:** Saif Khairat, Paige Ottmar, Betsy Sleath, Brandon Welch, Suparna Qanungo, Michelle Nichols, Jihad S Obeid

**Affiliations:** 1 University of North Carolina- Chapel Hill Chapel Hill, NC United States; 2 Medical University of South Carolina Charleston, SC United States

**Keywords:** telemedicine, informed consent, clinical trials, mobile phone

## Abstract

**Background:**

Informed consent is among the biggest challenges in recruiting participants for clinical research studies. Researchers face many challenges in conducting clinical trials, some of which include budgetary restrictions, lack of trained personnel, and difficulty recruiting study participants—particularly minorities and participants from rural communities.

**Objective:**

The objective of this study is to utilize telemedicine to improve the informed consent process for clinical trials and studies. We aim to assess the feasibility and efficacy of the teleconsent intervention among residents in urban and rural settings.

**Methods:**

This study will be conducted separately yet concurrently at two institutions, the Medical University of South Carolina and the University of North Carolina at Chapel Hill, to compare results within and across institutions.

**Results:**

Enrollment for Phase 1 began in March of 2018 and concluded in May 2018. Data transcription and analysis will be conducted through June and September of 2018.

**Conclusions:**

In this paper, we present a novel approach for conducting informed consent using a new telemedicine modality, namely, teleconsent. Teleconsent presents the ability to conduct a live interaction among clinical research coordinators and potential participants while synchronously presenting the consent form on the screen and obtaining participant’s signature through doxy.me, the teleconsent system. Teleconsent provides potential to improve obtaining informed consent from potential clinical trial participants.

**Registered Report Identifier:**

RR1-10.2196/11239

## Introduction

### Challenges Obtaining Consent for Research

Researchers face many challenges in conducting clinical trials, some of which include budgetary restrictions, lack of trained personnel, and difficulty recruiting study participants—particularly underserved and participants from rural communities [1]. Failure to meet enrollment goals can lead to a considerable amount of research waste, including costly time extensions [2,3], underpowered study results, and unpublished results as well as study termination and costing research institutions and sponsors a substantial investment in both money and time every year. Moreover, an ongoing concern in clinical trials is the typical underrepresentation of underserved individuals of a population [4], some of whom may lack the means for transportation to the study site but may have access to mobile devices, smartphones, or community centers (eg, public libraries or coffee shops) where the internet and computers are available [5]. This underrepresentation of underserved individuals could hinder the generalizability of study results and the translation of knowledge and potentially life-saving interventions into the routine clinical practice [4,6-9]; therefore, innovative approaches to increase access, improve participants’ experience, and increase trust to research studies are needed [10-12].

Multisite and offsite clinical trials often impose considerable travel costs and time commitments on participants [13]. The consenting process is often performed in-person with participants visiting with study personnel before, during, or after their clinical visit. In multisite clinical trials, consent often requires faxing or mailing of documents to the coordinating site [12]. In addition, training of clinic staff to maintain the regulatory compliance can add to the burden of study participation. Finally, as direct-to-patient recruitment sites and other nontraditional, novel recruitment approaches increase in popularity [14], the current informed consent process will be difficult, if not impossible, to scale. Informed consent readability and comprehension continue to be a major issue; a participant’s understanding during the consent process cannot easily be assessed by telephone or obtaining consent through an Web-based form [15,16]. The growing complexity of clinical studies and trials and the need to integrate trials into routine care (ie, the national movement toward more pragmatic clinical trials supported by the National Institutes of Health and the Patient-Centered Outcomes Research Institute, in particular) demand more accessible solutions for eliciting consent [17].

### Teleconsent as a Solution

**Figure 1:**
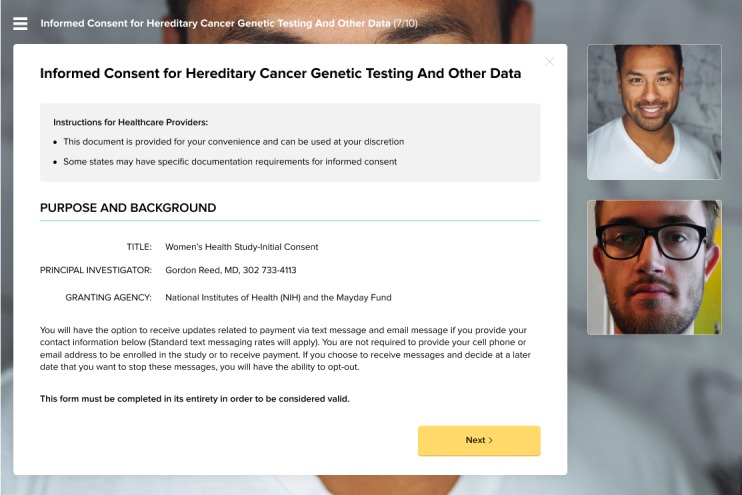
A mock consent form displayed in the Doxy.me software during a live teleconsent session.

The Biomedical Informatics team at the Medical University of South Carolina (MUSC) has developed “teleconsent,” an innovative, informed consent approach that leverages telemedicine technology to conduct remote, live consent sessions between participants and researchers. Teleconsent allows research personnel to meet and discuss the study with a prospective participant virtually using a video feed, share an informed consent document that can be collaboratively filled out by a participant and study personnel in real time, and generate an electronically signed informed consent document (Figure 1) available for immediate PDF download or print by both parties. This process can eliminate the inefficiencies related to travel, time, and management of personnel at remote sites [18]. In addition, the addition of a telehealth session will provide visual cues that may help research staff evaluate a potential participant’s understanding of risks, benefits, and other important elements of consent. New e-consent technologies may overcome some of the challenges related to comprehension by adding multimedia and interactive sessions testing participants’ comprehension [19]. To these tools, teleconsent adds opportunities for enhanced communication and remote access. Preliminary results, pilot studies, and anecdotal feedback regarding teleconsent have shown that participants are generally highly satisfied with teleconsent and did not experience difficulties understanding and navigating teleconsent and the process itself; however, there was a moderate, inverse relationship between age and satisfaction [20].

### Need for This Research

We are conducting a study that evaluates participants’ reactions to the teleconsent software, including their feelings regarding privacy and ease of use. We have 3 main objectives. First, we seek to understand the feasibility of using telehealth in rural areas, acceptability among underserved populations, and ethical and privacy concerns of using the teleconsent software and remote communication through participant interviews, particularly among minorities, rural, and elderly participants aged >65 years. Second, we aim to evaluate the quality of informed consent by comparing participants’ perception and comprehension using either teleconsent or paper-based consenting in ongoing clinical studies, through partnerships with investigators conducting those studies, by assigning participants to either teleconsent or traditional paper-based consenting. In addition, we will determine to what extent, if any, the teleconsent process affects participants’ informed consent and the workflow of research staff conducting the studies. Third, we intend to assess the workload on research assistants and their perception of the usability of teleconsent in terms of ease of use and user friendliness of the interface.

## Methods

### Study Setting

Study procedures will be conducted concurrently at 2 institutions, the MUSC and the University of North Carolina at Chapel Hill (UNC) to compare results both within and across institutions.

### Study Overview

**Figure 2:**
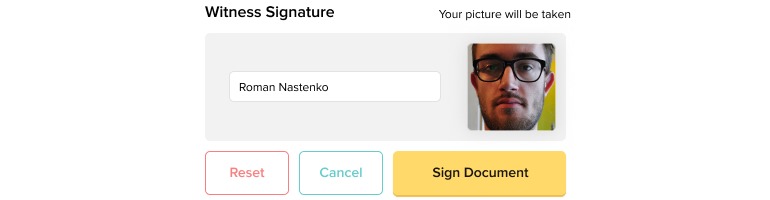
The teleconsent software allows the research coordinator and participant to sign the consent form in real time electronically.

This study is divided into 2 phases, to be completed in subsequent order and concurrently at both study sites. Phase 1 aims at meeting the first objective by conducting remote mock consent sessions using the Doxy.me software (Figures 1 and 2) and through interviewing participants about their experiences, preferences, and concerns with the technology, processes, and privacy.

In the second phase, we will complete our second and third objectives by partnering with ongoing clinical trials and assigning participants to either teleconsent or traditional paper-based consenting and then assessing the quality of informed consent and workload, among other metrics; of note, this part of the study will be initiated in the winter of 2019. Full procedures for both study parts are outlined in the “Procedures” section of this paper.

### Eligibility Criteria

For phase 1, prospective participants are required to be, at least, 18 years of age, currently residing in North or South Carolina, speak English as their first language, and have access to the internet and a computer with a microphone and camera. Any participant who does not meet the inclusion criteria will be excluded from the study. Our decision to exclude participants who do not reside in North Carolina is because one goal of this software is to study how North Carolina residents react to the software and compare the data to that of our MUSC. In addition, we choose to exclude participants who do not speak English as their first language to control for potential language barriers in this feasibility study. Moreover, the technological requirements of Doxy.me require participants to have access to a laptop or desktop with the appropriate audio-video capabilities; notably, the software was not compatible with tablets, such as iPads, or smartphone at the time of this publication. For phase 2, the eligibility criteria for our study is the same as those for the clinical trial sites.

### Recruitment

Each study site will have a unique recruitment process. For phase 1, in South Carolina, participants will be recruited through the dissemination of Institutional Review Board flyers placed in clinical and community settings and distributed by our Community Advisory Board members, advertisements, word-of-mouth, and at community-based clinic sites. Participants at UNC will be recruited through a researcher-participant networking website called Join the Conquest, word-of-mouth, and direct participant recruitment at Broad Street Clinic in Morehead, North Carolina. In addition, participants’ recruitment at UNC will be facilitated by 2 research assistants, the principle investigator and coprinciple investigator, and the staff at Broad Street Clinic. At MUSC, recruitment will be facilitated by the research coordinator. Both institutions will implement a prescreening eligibility questionnaire. On Join the Conquest, persons interested in the study will be directed to a Qualtrics eligibility survey, which will include demographic information. At MUSC, surveys will be completed through the Research Electronic Data Capture Platform (REDCap) [21]. From the pool of prospective participants obtained, we will select a total of 40 through a screening process that will be detailed later. Chosen participants will be offered gift cards or cash as remuneration.

For phase 2, participants will not be directly recruited for our study. Instead, we will partner with investigators conducting ongoing clinical research and assign those study participants to use either teleconsent or traditional paper-based consenting for the informed consent process. In total, 64 patients and up to 10 research assistants will be involved in this part of the study at the UNC site, though the number of research assistants may be as low as 2. Patients will be paid each time a set of surveys are completed. Research assistants will be paid upon the completion of each National Aeronautics and Space Administration (NASA)-Task Load Assessment (TLX) form (to be completed after each consent session) and also upon the completion of each System Usability Scale (SUS; to be completed approximately 3 times throughout the study). In addition, research assistants will be paid for participation in a focus group.

### Screening

For phase 1, participants’ screening will occur through the Qualtrics or REDCap surveys or over the phone. The demographic information collected during the screening process (age, gender, race, ethnicity, and county) will be used to select a group of participants who are coming from a variety of backgrounds to study potential differences between reactions to teleconsent among various demographic groups. For phase 2, participant screening will not occur.

### Randomization

No randomization will be involved in phase 1. For phase 2, if the parent study permits, participants will be either randomly assigned to each of teleconsent or traditional consent. When random assignment is not possible, for example, in studies in which some participants do not have the option of being assigned to traditional consent, thereby negating random assignment, we will use the propensity score matching after assignment to either teleconsent or traditional consent. Once verbally consented through one of these means, participants will undergo the informed consent process.

### Procedure

In phase 1, following the initial eligibility screening and identification as possible participants, interested individuals will be contacted by emails or phone to invite them to schedule a 1-hour mock consent session with a member of the study team. Before the mock consent session, participants at UNC will e-sign an Web-based consent form sent by Qualtrics and those at MUSC provide verbal consent at the time of scheduling, which is confirmed by the coinvestigator prior to participating in study procedures. During the mock consent session, study personnel will give participants a brief overview of Doxy.me and teleconsent before walking participants through a mock consent form created for this study. The purpose of the mock consent form is not to actually complete an informed consent session but to highlight the functionalities of the software. Immediately after the completion of the mock consent process, study personnel will conduct audiorecorded, semistructured interviews with participants about their experiences. After the study completion, participants will either receive cash or a gift card sent electronically or mailed to their residence(s). In total, 40 participants are to complete phase 1 of the study at each site, for a total of 80 participants between UNC and MUSC.

For phase 2, participants (n=64 at each site) will be assigned to either consenting for the clinical trial through teleconsent (n=32) or through paper-based consenting (n=32). Notably, 64 subjects per site (totaling n=128 between sites) will provide 80% power to detect modest differences (effect sizes ~0.50) between groups with respect to the survey instrument scores. After consenting for participation in the teleconsent study, participants will undergo the informed consent process using either teleconsent or traditional consent. After the completion of the consent process, participants will immediately fill out the quality of informed consent (QuIC), Decision-Making Control Instrument (DMCI, and Short Assessment of Health Literacy-English version (SAHL-E). Thirty days postconsent, participants will be asked to fill out this set of surveys once more electronically; they will be paid for each set of surveys completed.

Study personnel who are consenting potential participants will fill out a NASA-TLX form for each patient; they will be paid for each form filled. In addition, they will fill out SUS 3 times throughout the study, once at the beginning of their participation, once toward the middle, and once after they have completed enrolling participants for the teleconsent study; they will be paid for each SUS filled out. Finally, after the termination of the teleconsent partnership, those who will be obtaining informed consent for these studies and will assist in the collection of instruments will be invited to participate in a focus group asking about their workload and perceptions of the Doxy.me software for teleconsent. Focus group participants will be receiving remuneration for completing the focus group.

### Study Instruments

We will use the cognitive interviewing technique of verbal probing adapted from Willis to evaluate comprehension, decisions, and voluntariness, as proxies for users’ trust and intention to complete trials using the DMCI [22] and the QuIC [23] tool.

It is important to evaluate the decisional capacity and comprehension in informed consent processes, and research has identified that individuals with lower educational levels, mental illness, and advanced age are at risk of lower comprehension and potentially can be misled regarding the intent of the research [24]. To evaluate this, we will use the DMCI, a validated instrument that has been used to assess voluntary consent. The DMCI has a demonstrated internal consistency of 0.83 in psychometric studies [25]. The 9-item DMCI is used to assess perceived voluntariness, trust, and decision self-efficacy; in addition to a total score, it contains subscales addressing self-control, absence of control, and others’ control. In addition, the QuIC, a validated instrument that measures subjects’ understanding of the consent process in clinical trials and therapeutic misconception [23], is used to assess comprehension. The QuIC was designed to measure the actual (objective=20 items) and perceived (subjective=9 items) understanding of cancer clinical trials and can act as a screen for disclosure and capacity. The intraclass correlation coefficients (test-retest reliability) of.77 have demonstrated the reliability of this tool. We use the SAHL-E tool to measure health literacy. SAHL-E is an 18-item instrument that includes distractors on various health items, takes approximately 2 minutes to administer, and has a reliability of.89 [25].

In addition, validated surveys will be provided to all study personnel engaged in this process at the clinical trial sites to evaluate their level of satisfaction and workload experienced (NASA-TLX Task Index Scale) with the recruitment process [26,27]. NASA-TLX is a subjective workload assessment tool, which allows users to perform subjective workload assessments on operators working with various human-machine systems [26,27]. Using various metrics, including the QuIC tool [22], DMCI [23], SUS, the Doxy.me system through the NASA-TLX, SUS, and focus groups.

### Study Design

The design of phase 1 of our study is a qualitative study with a semistructured interview format. The ethical approval was obtained from the Institutional Review Boards at the UNC (17-2769) and the MUSC (# Pro00068715).

The design of phase 2 of our study is a 2-arm, teleconsent study, which assesses various metrics using standardized surveys. The ethical approval was obtained from the Institutional Review Board at the UNC (17-2870).

### Data Management

Survey assessments and demographics will be collected through UNC Qualtrics survey, REDCap survey, or pen-and-paper. Interview audios will be recorded using a handheld device (following participants’ consent) and stored on a secure MUSC Box server.

### Data Analysis

In phase 1, all interviews will be professionally transcribed and imported into the NVivo 12.0 (QSR International Pty, Doncaster, VIC, Australia) qualitative analysis software. Verification of the transcript accuracy will be performed prior to analyzing the text. An iterative process will be used in the analysis of the data. Then, 2 team members will code the data following its transcription by tagging segments of text in the transcripts to a concept, expanding, refining, and reducing the concepts, and discussing the findings in detail to allow for cross-validation of findings between the 2 sites [28]. A coinvestigator at MUSC with advanced training in research ethics and bioethics will explore the ethical appropriateness and associated principles of teleconsent using an integrative bioethical approach, and the MUSC research team will review emerging findings at the midpoint and conclusion of coding to consider what additional data will be necessary to refine our understanding of the participants’ perspectives related to teleconsent and determine overall themes. In addition, any feedback about the Doxy.me software will be submitted to developers at MUSC.

In phase 2, for patient participants, descriptive statistics (means, SDs, medians, etc) of responses to the survey instruments will be used to characterize the 2 groups (ie, teleconsent vs paper-based consent). Participants will be stratified with respect to demographics and clinical study. Each of the survey instrument summary scores and subscale scores will be essentially continuous variables; as such, comparisons between groups will be delineated through analysis of covariance modeling. In these models, the instrument score will serve as the dependent variable, with the experimental group serving as the key independent variable of interest. The analysis of covariance model includes participant factors, such as education and other demographics, as covariates in the models.

For study personnel, descriptive statistics will be used to study the differences in the NASA-TLX between the 2 groups through a 2-sample *t* test of means. The sample size of the research assistant group will be based on the number of research assistants currently working on the clinical trials. Regardless, we will assume 2-sided hypothesis testing with an alpha level of.05. We suspect that the null hypothesis will display no significant difference in the means of the research assistants’ NASA-TLX scores for teleconsent versus paper-based consenting.

## Results

### Timeline

Enrollment and data collection for phase 1 is expected to conclude by December 2018. Data transcription and analysis will begin in January 2019. For phase 2, 2 clinical trials have been identified for a potential partnership, and these sites have modified their Institutional Review Board protocols to include the addition of teleconsent. Phase 2 will also begin in January 2019. We anticipate reporting results in June 2019 through professional presentations and publications. Study findings will be disseminated through publications, direct update to Community Board Members, and electronically to participants.

### Dissemination

The results of the work from the above aims will be disseminated, throughout the Clinical Translational and Science Award (CTSA) consortium and the broader translational research community, through presentations at national meetings, such as the American Medical Informatics Association Translational Science Summit, relevant CTSA domain task force or interest group meetings, and through publication in peer-reviewed journals. The initial strategy for teleconsent is dissemination through academic research organizations in regional MUSC collaborations, including the Carolinas Collaborative and Mid-South Clinical Data Research Network. The teleconsent underlying framework, Doxy.me is a freely available lightweight telemedicine framework, which will facilitate the dissemination across the CTSA network.

## Discussion

Telemedicine is an innovative health care delivery model that provides care to patients at a distance using telecommunications capabilities [29]. Telemedicine has gained substantial support in recent years as an acceptable care methodology, with effective utilization in many clinical domains that has the potential to overcome several gaps and barriers in clinical trial enrollment by having the ability to remotely recruit and consent potential research participants, especially rural participants who are outside the proximity of the study team. Thus, teleconsent offers a convenient and complementary solution for researchers to meet with prospective participants and obtain consent. Researchers at the MUSC, partnered with the UNC, are interested in building off preliminary research focusing on the teleconsent software, Doxy.me, through a 2-part study.

In phase 1 of this study, participants will be recruited; participants will be residents of South and North Carolina (N=80; 40 from each respective site) from various backgrounds; however, all will meet the inclusion criteria specific to this study. Participants will be walked through the teleconsent process using a mock consent form and then interviewed to determine the overall themes regarding issues such as software difficulties and privacy issues. Results from this part of the study will be used to provide feedback to developers at the MUSC and address potential issues before phase 2, which will be completed in the summer of 2019. In addition, it will provide data on participant preferences, acceptability, and potential barriers to the adoption of teleconsent. For phase 2, researchers partner with ongoing clinical trials and assign participants (n=64 at each site) to either consenting by teleconsent or traditional paper-based consenting. Various validated surveys will be given to participants both immediately after the consent process and 30 days postconsent to determine differences in the understanding of the consent process between groups. Moreover, surveys will be administered to the study personnel who are consenting patients throughout the process to study the additional workload and demand placed upon them. We hypothesize that there will be no significant difference in the quality of informed consent or additional workload demand placed on researchers between traditional paper-based consenting and teleconsent. With these results, we hope to increase the usage of teleconsent in clinical trials to reduce barriers to study enrollment and improve underserved groups’ participation in research.

## Appendix

Multimedia Appendix 1 Notice of Award for Grant funding from NIH.

Multimedia Appendix 2 Summary statement and peer-review comments.

## Abbreviations

CTSA: Clinical Translational and Science Award
DMCI: Decision-Making Control Instrument
MUSC: Medical University of South Carolina
NASA: National Aeronautics and Space Administration
QuIC: Quality of Informed Consent
REDCap: Research Electronic Data Capture Platform
SAHL-E: Short Assessment of Health Literacy-English version
SUS: System Usability Scale
TLX: Task Load Assessment
UNC: University of North Carolina at Chapel

## References

[1] Sung N, Crowley William F, Genel Myron, Salber Patricia, Sandy Lewis, Sherwood Louis M, Johnson Stephen B, Catanese Veronica, Tilson Hugh, Getz Kenneth, Larson Elaine L, Scheinberg David, Reece E Albert, Slavkin Harold, Dobs Adrian, Grebb Jack, Martinez Rick A, Korn Allan, Rimoin David (2003). Central challenges facing the national clinical research enterprise. JAMA.

[2] Friedewald W (1990). Costs of clinical trials and the need for efficiency: a brief overview. Stat Med.

[3] Sharma S, Tripathi KD (2008). Essentials of Medical Pharmacology.

[4] Hamel L, Penner Louis A, Albrecht Terrance L, Heath Elisabeth, Gwede Clement K, Eggly Susan (2016). Barriers to Clinical Trial Enrollment in Racial and Ethnic Minority Patients With Cancer. Cancer Control.

[5] Glick HA, Doshi JA, Sonnad SS, Polsky D (2014). Economic evaluation in clinical trials.

[6] Sonne S, Andrews Jeannette O, Gentilin Stephanie M, Oppenheimer Stephanie, Obeid Jihad, Brady Kathleen, Wolf Sharon, Davis Randal, Magruder Kathryn (2013). Development and pilot testing of a video-assisted informed consent process. Contemp Clin Trials.

[7] Committee on Cancer Clinical Trials, The NCI Cooperative Group Program (2018). A National Cancer Clinical Trials System for the 21st Century: Reinvigorating the NCI Cooperative Group Program.

[8] Gul R, Ali Parveen A (2010). Clinical trials: the challenge of recruitment and retention of participants. J Clin Nurs.

[9] Campbell M, Snowdon C, Francis D, Elbourne D, McDonald A M, Knight R, Entwistle V, Garcia J, Roberts I, Grant A, Grant A, STEPS group (2007). Recruitment to randomised trials: strategies for trial enrollment and participation study. The STEPS study. Health Technol Assess.

[10] Pinsky P, Ford Marvella, Gamito Eduard, Higgins Darlene, Jenkins Victoria, Lamerato Lois, Tenorio Sally, Marcus Pamela M, Gohagan John K (2008). Enrollment of racial and ethnic minorities in the Prostate, Lung, Colorectal and Ovarian Cancer Screening Trial. J Natl Med Assoc.

[11] (2006). NIH policy and guidelines on the inclusion of women and minorities as subjects in clinical research: amended, October.

[12] Heller C, Balls-Berry Joyce E, Nery Jill Dumbauld, Erwin Patricia J, Littleton Dawn, Kim Mimi, Kuo Winston P (2014). Strategies addressing barriers to clinical trial enrollment of underrepresented populations: a systematic review. Contemp Clin Trials.

[13] English RA, Lebovitz Y, Giffin RB, Forum on Drug Discovery, Development, and Translation, Institute of Medicine, Board on Health Sciences Policy (2010). Transforming Clinical Research in the United States: Challenges and Opportunities: Workshop Summary.

[14] Fenner Yeshe, Garland Suzanne M, Moore Elya E, Jayasinghe Yasmin, Fletcher Ashley, Tabrizi Sepehr N, Gunasekaran Bharathy, Wark John D (2012). Web-based recruiting for health research using a social networking site: an exploratory study. J Med Internet Res.

[15] Reidenberg M (2005). Informed consent or acknowledgment of disclosure. Clin Pharmacol Ther.

[16] Fortun P, West J, Chalkley L, Shonde A, Hawkey C (2008). Recall of informed consent information by healthy volunteers in clinical trials. QJM.

[17] Hayden E (2012). Informed consent: a broken contract. Nature.

[18] Welch B, Marshall Elizabeth, Qanungo Suparna, Aziz Ayesha, Laken Marilyn, Lenert Leslie, Obeid Jihad (2016). Teleconsent: A Novel Approach to Obtain Informed Consent for Research. Contemp Clin Trials Commun.

[19] Rothwell Erin, Wong Bob, Rose Nancy C, Anderson Rebecca, Fedor Beth, Stark Louisa A, Botkin Jeffrey R (2014). A randomized controlled trial of an electronic informed consent process. J Empir Res Hum Res Ethics.

[20] Newlin T, McCall Terika, Ottmar Paige, Welch Brandon, Khairat Saif (2018). Assessing the Satisfaction of Citizens Using Teleconsent in Clinical Research. Stud Health Technol Inform.

[21] Harris PA, Taylor R, Thielke R, Payne J, Gonzalez N, Conde JG (2009). Research electronic data capture (REDCap)--a metadata-driven methodology and workflow process for providing translational research informatics support. J Biomed Inform.

[22] Miller V, Ittenbach Richard F, Harris Diana, Reynolds William W, Beauchamp Tom L, Luce Mary Frances, Nelson Robert M (2011). The decision making control instrument to assess voluntary consent. Med Decis Making.

[23] Joffe S, Cook E F, Cleary P D, Clark J W, Weeks J C (2001). Quality of informed consent: a new measure of understanding among research subjects. J Natl Cancer Inst.

[24] Flory J, Emanuel Ezekiel (2004). Interventions to improve research participants' understanding in informed consent for research: a systematic review. JAMA.

[25] Lee S, Stucky Brian D, Lee Jessica Y, Rozier R Gary, Bender Deborah E (2010). Short Assessment of Health Literacy-Spanish and English: a comparable test of health literacy for Spanish and English speakers. Health Serv Res.

[26] Harris Don, Chan-Pensley Jamie, McGarry Shona (2005). The development of a multidimensional scale to evaluate motor vehicle dynamic qualities. Ergonomics.

[27] Spector (1994). Job satisfaction survey. Tampa, Florida: Department of Psychology, University of South Florida (1994).

[28] Bradley E, Curry Leslie A, Devers Kelly J (2007). Qualitative data analysis for health services research: developing taxonomy, themes, and theory. Health Serv Res.

[29] What is Telemedicine.

