# Supplementary material for: Facilitating the Informed Consent Process Using Teleconsent: Protocol for a Feasibility and Efficacy Study
Source: JMIR Res Protoc. 2018 Oct 17;7(10):e11239. doi: 10.2196/11239 (PMC6234333; doi:10.2196/11239)
Supplement: Multimedia Appendix 1 [file resprot_v7i10e11239_app1.pdf]

EXPLORATORY/DEVELOPMENT GRANT **Federal Award Date:** 07/14/2017
Department of Health and Human Services
National Institutes of Health

NATIONAL CENTER FOR ADVANCING TRANSLATIONAL SCIENCES

Grant Number: 1R21TR002088-01
FAIN: R21TR002088

Principal Investigator(s):
Jihad Obeid, MD

Project Title: Investigating teleconsent to improve clinical research access in remote communities

Mr. McCants, R. Darren
Director
19 Hagood Ave. Suite 606
MSC 808
Charleston, SC 294258080

Award e-mailed to: orsp@musc.edu

Period Of Performance:
Budget Period: 08/01/2017 – 07/31/2018
Project Period: 08/01/2017 – 07/31/2019

Dear Business Official:

The National Institutes of Health hereby awards a grant in the amount of $238,125 (see “Award Calculation” in Section I and “Terms and Conditions” in Section III) to MEDICAL UNIVERSITY OF SOUTH CAROLINA in support of the above referenced project. This award is pursuant to the authority of 42 USC 241 42 CFR 52 and is subject to the requirements of this statute and regulation and of other referenced, incorporated or attached terms and conditions.

Acceptance of this award including the “Terms and Conditions” is acknowledged by the grantee when funds are drawn down or otherwise obtained from the grant payment system.

Each publication, press release, or other document about research supported by an NIH award must include an acknowledgment of NIH award support and a disclaimer such as “Research reported in this publication was supported by the National Center For Advancing Translational Sciences of the National Institutes of Health under Award Number R21TR002088. The content is solely the responsibility of the authors and does not necessarily represent the official views of the National Institutes of Health.” Prior to issuing a press release concerning the outcome of this research, please notify the NIH awarding IC in advance to allow for coordination.

Award recipients must promote objectivity in research by establishing standards that provide a reasonable expectation that the design, conduct and reporting of research funded under NIH awards will be free from bias resulting from an Investigator’s Financial Conflict of Interest (FCOI), in accordance with the 2011 revised regulation at 42 CFR Part 50 Subpart F. The Institution shall submit all FCOI reports to the NIH through the eRA Commons FCOI Module. The regulation does not apply to Phase I Small Business Innovative Research (SBIR) and Small Business Technology Transfer (STTR) awards. Consult the NIH website http://grants.nih.gov/grants/policy/coi/ for a link to the regulation and additional important information.

If you have any questions about this award, please contact the individual(s) referenced in Section IV.

Sincerely yours,

Gavin Wilkom
Grants Management Officer
NATIONAL CENTER FOR ADVANCING TRANSLATIONAL SCIENCES

Additional information follows

SECTION I – AWARD DATA – 1R21TR002088-01

Award Calculation (U.S. Dollars)

**Federal Direct Costs**	$181,200
**Federal F&A Costs**	$56,925
**Approved Budget**	$238,125
**Total Amount of Federal Funds Obligated (Federal Share)**	$238,125
**TOTAL FEDERAL AWARD AMOUNT**	$238,125
**AMOUNT OF THIS ACTION (FEDERAL SHARE)**	$238,125

SUMMARY TOTALS FOR ALL YEARS		
YR	THIS AWARD	CUMULATIVE TOTALS
1	$238,125	$238,125
2	$188,125	$188,125

Recommended future year total cost support, subject to the availability of funds and satisfactory progress of the project

Fiscal Information:
CFDA Name: National Center for Advancing Translational Sciences
CFDA Number: 93.350
EIN: 1576000722A2
Document Number: RTR002088A
PMS Account Type: P (Subaccount)
Fiscal Year: 2017

IC	CAN	2017	2018
TR	8014099	$238,125	$188,125

Recommended future year total cost support, subject to the availability of funds and satisfactory progress of the project

NIH Administrative Data:
PCC: CRT35 / **OC**: 414A / **Released**: WILKOMG 07/10/2017
Award Processed: 07/14/2017 12:22:17 AM

SECTION II – PAYMENT/HOTLINE INFORMATION – 1R21TR002088-01

For payment and HHS Office of Inspector General Hotline information, see the NIH Home Page at http://grants.nih.gov/grants/policy/awardconditions.htm

SECTION III – TERMS AND CONDITIONS – 1R21TR002088-01

This award is based on the application submitted to, and as approved by, NIH on the above-titled project and is subject to the terms and conditions incorporated either directly or by reference in the following:

a. The grant program legislation and program regulation cited in this Notice of Award.
b. Conditions on activities and expenditure of funds in other statutory requirements, such as those included in appropriations acts.
c. 45 CFR Part 75.
d. National Policy Requirements and all other requirements described in the NIH Grants Policy Statement, including addenda in effect as of the beginning date of the budget period.
e. Federal Award Performance Goals: As required by the periodic report in the RPPR or in the final progress report when applicable.
f. This award notice, INCLUDING THE TERMS AND CONDITIONS CITED BELOW.

(See NIH Home Page at http://grants.nih.gov/grants/policy/awardconditions.htm for certain references cited above.)

Research and Development (R&D): All awards issued by the National Institutes of Health (NIH) meet the definition of "Research and Development" at 45 CFR Part§ 75.2**.** As such, auditees should identify NIH awards as part of the R&D cluster on the Schedule of Expenditures of Federal Awards (SEFA). The auditor should test NIH awards for compliance as instructed in Part V, Clusters of Programs. NIH recognizes that some awards may have another classification for purposes of indirect costs. The auditor is not required to report the disconnect (i.e., the award is classified as R&D for Federal Audit Requirement purposes but non-research for indirect cost rate purposes), unless the auditee is charging indirect costs at a rate other than the rate(s) specified in the award document(s).

This institution is a signatory to the Federal Demonstration Partnership (FDP) Phase VI Agreement which requires active institutional participation in new or ongoing FDP demonstrations and pilots.

An unobligated balance may be carried over into the next budget period without Grants Management Officer prior approval.

This grant is subject to Streamlined Noncompeting Award Procedures (SNAP).

This award is subject to the requirements of 2 CFR Part 25 for institutions to receive a Dun & Bradstreet Universal Numbering System (DUNS) number and maintain an active registration in the System for Award Management (SAM). Should a consortium/subaward be issued under this award, a DUNS requirement must be included. See http://grants.nih.gov/grants/policy/awardconditions.htm for the full NIH award term implementing this requirement and other additional information.

This award has been assigned the Federal Award Identification Number (FAIN) R21TR002088. Recipients must document the assigned FAIN on each consortium/subaward issued under this award.

Based on the project period start date of this project, this award is likely subject to the Transparency Act subaward and executive compensation reporting requirement of 2 CFR Part 170. There are conditions that may exclude this award; see http://grants.nih.gov/grants/policy/awardconditions.htm for additional award applicability information.

In accordance with P.L. 110-161, compliance with the NIH Public Access Policy is now mandatory. For more information, see NOT-OD-08-033 and the Public Access website: http://publicaccess.nih.gov/.

In accordance with the regulatory requirements provided at 45 CFR 75.113 and Appendix XII to 45 CFR Part 75, recipients that have currently active Federal grants, cooperative agreements, and procurement contracts with cumulative total value greater than $10,000,000 must report and maintain information in the System for Award Management (SAM) about civil, criminal, and administrative proceedings in connection with the award or performance of a Federal award that reached final disposition within the most recent five-year period. The recipient must also make semiannual disclosures regarding such proceedings. Proceedings information will be made publicly available in the designated integrity and performance system (currently the Federal Awardee Performance and Integrity Information System (FAPIIS)). Full reporting requirements and procedures are found in Appendix XII to 45 CFR Part 75. This term does not apply to NIH fellowships.
Treatment of Program Income:
Additional Costs

SECTION IV – TR Special Terms and Conditions – 1R21TR002088-01

SUBJECT FOA

This award is subject to the conditions set forth in RFA -TR-16-343, “Limited Competition: Exploratory CTSA Collaborative Innovation Awards (R21),” which are hereby incorporated by reference as special terms and conditions of this award. Copies of this Funding Opportunity Announcement can be found at the following link: https://grants.nih.gov/grants/guide/pa-files/PAR-16-343.html

NCATS FUNDING PLAN FOR FY2017
This competing award reflects the NIH Fiscal Policy for Grant Awards for FY2017 (see NIH Guide Notice NOT-17-086) and the implementation of the NCATS FY2017 grants funding policy: https://ncats.nih.gov/funding/review/policy

CONSORTIUM
This award includes funds awarded for subcontractual/consortium activity with University of North Carolina at Chapel Hill in the amount of $91,200 total costs ($60,000 direct costs + $31,200 facilities and administrative costs). Consortia are to be established and administered as described in the NIH Grants Policy Statement (NIH GPS). The referenced section of the NIH GPS, Part II Chapter 15 is available at: http://grants.nih.gov/grants/policy/nihgps/nihgps.pdf

MODULAR GRANT
INFORMATION: This is a Modular Grant Award without direct cost categorical breakdowns issued in accordance with the guidelines published in the NIH Grants Policy Statement, see http://grants.nih.gov/grants/policy/nihgps/nihgps.pdf. Recipients are required to allocate and account for costs related to this award by category within their institutional accounting system in accordance with applicable cost principles.

KEY PERSONNEL
In addition to the PI, the following individual is named as key personnel (individuals who has effort that NCATS staff is tracking):

Saif Khairat

Written prior approval is required if the individual named above withdraws from the project entirely, is absent from the project during any continuous period of 3 months or more, or reduces time devoted to the project by 25 percent or more from the level that was approved at the time of award.

Non Key Personnel

Based on the other support submitted on 06/27/2017, the following personnel may be committed over 12 Calendar Months (CM) with the awarding of this grant,depending other pending support:

Betsy Sleath
Brandon Welch

The grantee institution is responsible for adjusting the effort as needed so that at no time the above named individual(s) total effort exceeds 12 CM.

NON-COMPETING RENEWAL (SNAP)
The NIH requires the use of the Research Performance Progress Report (RPPR) for all Type 5 progress reports. The RPPR and other documents applicable to this SNAP grant are due the 15th of the month preceding the month in which the budget period ends (e.g., if the budget period ends 11/30, the due date is 10/15). Please see http://grants.nih.gov/grants/rppr/index.htm for additional information on the RPPR.

PRIOR APPROVAL REQUEST
Any prior approval request (e.g., changes to key personnel as noted on the award, changes in human and animal subjects requiring prior approval, carryover requests) must be submitted to the assigned Grants Management Specialist and Programmatic Official. Please refer to Part II Chapter 8 the NIH Grants Policy Statement for the activities and/or expenditures that require NIH approval at http://grants.nih.gov/grants/policy/nihgps/nihgps.pdf

COMMUNICATIONS/PRESS RELEASE

If the grantee plans to issue a press release concerning the outcome of NCATS grant-supported research, it should notify the NCATS Office of Communications at 301-435-0888, in advance to allow for coordination.

The NCATS WWW home page is at http://ncats.nih.gov/

STAFF CONTACTS

The Grants Management Specialist is responsible for the negotiation, award and administration of this project and for interpretation of Grants Administration policies and provisions. The Program Official is responsible for the scientific, programmatic and technical aspects of this project. These individuals work together in overall project administration. Prior approval requests (signed by an Authorized Organizational Representative) should be submitted in writing to the Grants Management Specialist. Requests may be made via e-mail.

Grants Management Specialist: Laura Florette Gray
Email: laura.gray@nih.gov **Phone**: 301-451-4238

Program Official: Pj Brooks
Email: pjbrooks@mail.nih.gov **Phone**: 301-443-0513

SPREADSHEET SUMMARY
GRANT NUMBER: 1R21TR002088-01

INSTITUTION: MEDICAL UNIVERSITY OF SOUTH CAROLINA

Facilities and Administrative Costs	Year 1	Year 2
F&A Cost Rate 1	49.5%	49.5%
F&A Cost Base 1	$115,000	$75,000
F&A Costs 1	$56,925	$37,125
