# Supplementary material for: Facilitating the Informed Consent Process Using Teleconsent: Protocol for a Feasibility and Efficacy Study
Source: JMIR Res Protoc. 2018 Oct 17;7(10):e11239. doi: 10.2196/11239 (PMC6234333; doi:10.2196/11239)
Supplement: Multimedia Appendix 2 [file resprot_v7i10e11239_app2.pdf]

SUMMARY STATEMENT
(Privileged Communication)

PROGRAM CONTACT:
Dr. PJ Brooks
301-443-0513
pjbrooks@mail.nih.gov

Release Date: **04/04/2017**
Revised Date:

Application Number: **1 R21 TR002088-01**

Principal Investigator

OBEID, JIHAD

Applicant Organization: MEDICAL UNIVERSITY OF SOUTH CAROLINA

Review Group: **ZTR1 CI-4 (01)**
National Center for Advancing Translational Sciences Special Emphasis Panel
Collaborative Innovation Award (U01 and R21) Review Meeting

Meeting Date: **03/08/2017**
Council: **MAY 2017**
Requested Start: **09/01/2017**
RFA/PA: **PAR16-343**
PCC: **CRT35**

Project Title: **Investigating teleconsent to improve clinical research access in remote communities**
SRG Action: **Impact Score:35**
Next Steps: **Visit https://grants.nih.gov/grants/next_steps.htm**
Human Subjects: 30-Human subjects involved - Certified, no SRG concerns
Animal Subjects: 10-No live vertebrate animals involved for competing appl.
Gender: 1A-Both genders, scientifically acceptable
Minority: 1A-Minorities and non-minorities, scientifically acceptable
Children: 3A-No children included, scientifically acceptable
Clinical Research - not NIH-defined Phase III Trial

Project Year	Direct Costs Requested	Estimated Total Cost
1	150,000	232,500
2	125,000	193,750
TOTAL	275,000	426,250

ADMINISTRATIVE BUDGET NOTE: The budget shown is the requested budget and has not been adjusted to reflect any recommendations made by reviewers. If an award is planned, the costs will be calculated by Institute grants management staff based on the recommendations outlined below in the COMMITTEE BUDGET RECOMMENDATIONS section.

1R21TR002088-01 Obeid, Jihad

RESUME AND SUMMARY OF DISCUSSION: This is a new application for the Limited Competition: Exploratory CTSA Collaborative Innovation Award (R21) from the Medical University of South Carolina entitled "Investigating teleconsent to improve clinical research access in remote communities."

This application addresses the important issue of obtaining informed consent using teleconsent via a combination of online forms and video conferencing for translational research and other research projects. The strengths of this application include the ability to recruit patients from remote areas, an investigator who is experienced with the proposed intervention, the implementation and adoption of technology to reduce travel burden on participants as well as regulatory burden on clinical investigator, and a study that is likely to yield useful information on the intervention approach. While this study is carefully designed, there are a number of perceived limitations. Some of the weaknesses include little involvement of participants, communities and families in the design, management and interpretation of the study. There is also lack of discussion or presentation of information on previous attempts to address teleconsent and whether or not this is a particularly innovative approach, along with some concern about the ability or utility of the instruments that are being used. Other concerns include how the project would be managed within and across the sites, perhaps related to the suggestion for additional senior involvement, and a lack of discussion as to how the investigators will identify and manage the inability of participants to provide consent. There were some differences of opinion regarding whether a truly participatory study would be difficult to implement within the time frame, and whether the use of a mixed methods approach allows sufficient feedback from participants. In summary, despite the strengths of the application, the number of weaknesses slightly tempered the reviewer's enthusiasm for this application.

Overall, the application received an Impact/Priority Score of 35; the committee recommended the budget as requested.

DESCRIPTION (provided by applicant): Recruitment and enrollment of eligible research participants into clinical trials is a major challenge in most clinical settings, including informed consent at remote sites. Studies often fail to meet enrollment goals, resulting in costly time extensions, underpowered results, and in some cases early termination. Informed consent is an essential process involving trained research personnel meeting face-to-face with participants, which can be especially challenging during busy clinic schedules or recruitment at remote locations. An innovative informed consent approach that leverages telemedicine technology (teleconsent) was developed at the Medical University of South Carolina (MUSC). Teleconsent allows research personnel to: 1) meet and discuss the study with a prospective participant virtually using a video feed; 2) share an informed consent document that can be collaboratively filled out by participant and personnel in real-time; and 3) generate an electronically signed informed consent that is available for immediate download or print by both parties. The objective of this proposal is to evaluate teleconsent in real-world environments across two institutions, MUSC and the University of North Carolina at Chapel Hill. This includes the examination of ethical and privacy concerns by stakeholders and the community, and identifying barriers to adoption. The aims are to: 1) evaluate the feasibility, ethics, and impact of teleconsent on access at remote sites including underserved communities and on informed consent comprehension; 2) assess the usability of the technology and its impact on the research workflow, both at local (coordinating center or researcher's home institution) as well as at remote locations (remote clinics or other recruitment facilities). If successful, this work will show the utility of this new technology, identify potential barriers to adoption and inform implementation in other research environments. A positive outcome should provide an avenue to improve recruitment/enrollment rates, reduce the burden associated with obtaining regulatory approval for remote sites, lower the costs of remote enrollment, and extend research into underserved areas, without negatively impacting the informed consent process.

PUBLIC HEALTH RELEVANCE (provided by applicant): The objective of this proposal is to evaluate teleconsent, a novel telemedicine informed consent system, in order to study the advantages of teleconsent, the barriers to its adoption, and its impact on the informed consent process. The goal of this work is to improve the adoption of this technology and improve the overall research process, with reduction in travel burden on research participants and regulatory burden on clinical investigators. Facilitating enrollment into clinical trials will in turn accelerate the development of new treatments.

CRITIQUE

Critique 1

Significance: 4
Investigator(s): 2
Innovation: 5
Approach: 4
Environment: 2

Overall Impact

This project focuses on deploying and evaluating teleconsent for remote patients for clinical trials. This study is carefully designed with the aims of evaluating the teleconsenting approaches of patients using a combination of online forms and video conferencing based consulting and the impact on clinical workflow. The overall design is carefully considered including sample size and the justification on the consortium is solid. However, what dampens the enthusiasm is that the idea of remote consent is not new and has apparently been considered by the Food and Drug Administration (FDA) for years.

Significance

Strengths

- The scientific premise of this project is sound as there is a need for increasing the participation and access to clinical trials for patients living in remote areas. The consenting and consulting for these patients are usually difficult and teleconsenting, taking advantage of Information Technology (IT) development, is an alternative choice which can potentially improve the delivery of advanced health care to these patients.
- The patients covered by these two CTSA institutes have a higher than average underrepresented patient groups living in remote areas, and there is a need for improving health care disparity using IT technologies.

Weaknesses

- Since there have been a number of studies on remote consent using various mobile applications, it is not clear what new insight is going to be gained from this study.

Investigator(s)

Strengths

- Dr. Jihad Obeid, M.D. is a well-known expert on biomedical informatics.

- This is a comprehensive team including all the expertise and stakeholders.

Weaknesses

- None.

Innovation

Strengths

- Teleconsent using the Doxyme application.

Weaknesses

- There have been many studies on remote consent and an evaluation report (a PubMed search leads to 252 entries with the newest one being one for formative evaluation of mobile eConsent using an application). So the concept is not innovative.

Approach

Strengths

- The study is carefully designed and rigorous with a statistically justified cohort size, balanced gender and ethnicity.
- The study covers not only evaluation on patient's experience, but also appraises clinical workflow using clear evaluation methods.

Weaknesses

- According to the Institutional Review Board (IRB), all the participants need to complete IRB-approved consent.

Environment

Strengths

- The environment in Medical University of South Carolina (MUSC) and the University of North Carolina (UNC) is adequate for this project.

Weaknesses

- None.

Protections for Human Subjects

Acceptable. The IRB is consulted and a clear guideline is given. According to the IRB rules, all the participants need to complete IRB-approved consent. But since the test itself is on consenting, it is curious how this loop cannot be broken.

Inclusion of Women, Minorities and Children and not IRB Exemption #4.

- Sex/Gender: Distribution justified scientifically.
- Race/Ethnicity: Distribution justified scientifically.
- Inclusion/Exclusion of Children under 18: Excluding ages <18; justified scientifically.
- The inclusion and distributions are well justified.

Resource Sharing Plans

Acceptable.

Budget and Period of Support

Recommend as Requested.

Critique 2

Significance: 1

Investigator(s): 1

Innovation: 2

Approach: 1

Environment: 1

Overall Impact

The purpose of this study is to evaluate teleconsent, a telemedicine informed consent system, to study the advantages of teleconsent, barriers to adoption, and impact on the informed consent process. Overall, the longer term goal is to improve the adoption of this technology, overall research process, and reduce travel burden on research participants and regulatory burden on clinical investigators. This is an innovative concept and the exploratory aims are appropriate to test this new technology and process. The methods are well conceptualized, the analyses section is strong, and the actual teleconsent has multiple modalities. This is a thoughtful application lead by an experienced team and has high potential for impact.

Significance

Strengths

- Using telemedicine to obtain consent could increase access to clinical trials, reduce travel burden on staff and patients.
- Results could be implemented across CTSA.

Weaknesses

- None noted.

Investigator(s)

Strengths

- The team has significant experience in telemedicine, e-consent, and developing such platforms.

Innovation

Strengths

- Leverages the benefits of telemedicine technology increase access, reach, and enrollment into clinical trials.

Weaknesses

- None noted.

Approach
Strengths

- Well conceptualized methods and analysis plans and a very well written application.
- Feasibility and usability assessments are well-described.
- Considerations of ethics for using teleconsent in underserved populations.
- Assessment of impact on workflow etc., will add significant information to the area.
- The use of observation and self-report for outcomes and mixed data (qualitative and quantitative) is a strength.
- The e-consent itself takes into account multiple learning needs (video and audio not just text).

Weaknesses

- Because the aims are formative in nature, there may not be a need to randomize to paper or teleconsent groups; however, this is merely a comment not a weakness, per se.

Environment
Strengths

- Excellent environment at MUSC.

Weaknesses

- None noted.

Protections for Human Subjects
Acceptable Risks and/or Adequate Protections. This is an interesting concept in that informed consent is required to test a consent format. However, participants will be asked to sign consent.

Inclusion of Women, Minorities and Children and not IRB Exemption #4.

- Sex/Gender: Distribution justified scientifically
- Race/Ethnicity: Distribution justified scientifically
- Inclusion/Exclusion of Children under 18: Excluding ages <18; justified scientifically.
- All inclusions and exclusions are appropriate for this study.

Resource Sharing Plans
Acceptable.

Budget and Period of Support:
Recommend as Requested.

Critique 3

Significance: 3
Investigator(s): 3
Innovation: 3

Approach: 4

Environment: 1

Overall Impact

MUSC and the UNC CTSA hubs are requesting support for an evaluation of teleconsent procedures for clinical trials developed and currently in limited use at MUSC. The teleconsent procedures allow research personnel to use video feeds to meet and discuss a study with prospective participants; share, review, discuss and complete an informed consent document in real time; and generate an electronically signed consent document. This application addresses an important issue in obtaining informed consent for translational and other research projects. The applicant has some experience with the proposed intervention, multi-methods are appropriately used in the evaluation, and the study is likely to yield useful information on the intervention approach. However, there are also limitations that restrict the potential impact. First, there is little involvement of participants, communities, and families in the design, management, and interpretation of the study. The project would benefit from some senior oversight. There is little discussion of how the investigators will identify and manage participant inability to provide consent. It would also have been helpful if there is more clarity in identifying circumstances or populations with whom the approach is likely to be most appropriate.

Significance

Strengths

- This project addresses part of the problem in recruiting and retaining patients in clinical trials, particularly multi-site trials with potential and actual participants at some distance from trial sites.
- Theoretically at least, successful completion of the study will provide an alternative to face-to-face participant enrollment.
- The applicant will make the platform and procedures available for use at other CTSA hubs.

Weaknesses

- While the results of this study may increase participation in studies in rural and isolated sites, it is not clear how the results may serve to increase participation among minority and disenfranchised communities.
- While there are provisions for dissemination, interventions like this may require technical, training, and other types of support for dissemination to other settings. It is not clear how this would occur.
- It is not at all clear how they will identify and manage potential participants who are not able to provide consent.

Investigator(s)

Strengths

- The PI, Co-Is and staff appear generally well qualified; although the PI is an Associate Professor and all of the Co-Is at MUSC are Assistant Professors. The study might benefit from a more senior oversight committee or advisory committee.

Weaknesses

- There is little information on exactly how the project will be managed and coordinated within MUSC, within UNC, and across the two institutions.
- Dr. Nichols has appropriate experience in Community-Based Participatory Research (CBPR), health literacy and related areas, but it is not clear from her vitae or narrative how much

experience she has directly related to the qualitative interviewing and analysis tasks in Sub-aim 1 and Sub-aim 2, or in mixed methods.

Innovation

Strengths

- The inclusion of qualitative, quantitative and mixed methods in this study is a very positive aspect of the design.

Weaknesses

- The applicant provides some information on the specific instruments used in Sub-aim 1b. However, internal consistency measures actually do not seem to say very much about how well the instrument represents or measures the underlying concepts.
- It would be particularly useful to have more information on how effective the Quality of Informed Consent is in identifying individuals who are not able to provide informed consent.
- There is a lack of information on other projects that have addressed this issue, and how innovative this approach is.

Approach

Strengths

- The overall approach is appropriate as first steps in validating this approach to informed consent. Alternative approaches to dealing with barriers are discussed.
- The project is very likely to provide useful information. However, it is not completely clear how they will know if their approach is successful.
- While the applicant notes that this approach is being used and considered for a few projects at MUSC, little information is provided on issues related to the strategy.
- There is limited discussion of dissemination of the proposed approach, and the applicant does agree to make the platform available.

Weaknesses

- There is little in the way of participant, family, and community engagement in this project, except to provide information. Given that informed consent may be important to these groups, the project should incorporate some type of participant and family advisory groups to provide input and oversight into the project.
- It is interesting that there is no consideration in this project to issues related to community consent such as it might be required on Native American reservations and as increasingly discussed in the community literature on CBPR.
- It would be helpful if the applicant devoted some attention to identifying when or with which populations this approach is likely to be successful.

Environment

Strengths

- The environment is appropriate and the applicant has some experience with the approach.

Weaknesses

- None noted.

Protections for Human Subjects

Acceptable Risks and/or Adequate Protections.

Inclusion of Women, Minorities and Children and not IRB Exemption #4.

- Sex/Gender: Distribution justified scientifically.
- Race/Ethnicity: Distribution justified scientifically.
- Inclusion/Exclusion of Children under 18: Excluding ages <18; justified scientifically.

Resource Sharing Plans

Acceptable. It is not clear from the discussion on resource sharing whether the actual data collected through the study will be available outside the research group(s).

Budget and Period of Support

Recommend as Requested. There is some confusion about the total and direct cost amounts listed on page 70. Are these within the range specified in the RFA?

Additional Comments to Applicant (Optional)

This is a difficult application to read and understand. It would have been very helpful to have a single table that describes the subjects, measures, and purpose of each of the sub-studies.

THE FOLLOWING SECTIONS WERE PREPARED BY THE SCIENTIFIC REVIEW OFFICER TO SUMMARIZE THE OUTCOME OF DISCUSSIONS OF THE REVIEW COMMITTEE, OR REVIEWERS' WRITTEN CRITIQUES, ON THE FOLLOWING ISSUES:

PROTECTION OF HUMAN SUBJECTS (Resume): ACCEPTABLE

INCLUSION OF WOMEN PLAN (Resume): ACCEPTABLE

INCLUSION OF MINORITIES PLAN (Resume): ACCEPTABLE

INCLUSION OF CHILDREN PLAN (Resume): ACCEPTABLE

COMMITTEE BUDGET RECOMMENDATIONS: The budget was recommended as requested. There is some confusion about the total and direct cost amounts listed on page 70. Are these within the range specified in the RFA?

Footnotes for 1 R21 TR002088-01; PI Name: Obeid, Jihad

NIH has modified its policy regarding the receipt of resubmissions (amended applications). See Guide Notice NOT-OD-14-074 at http://grants.nih.gov/grants/guide/notice-files/NOT-OD-

14-074.html. The impact/priority score is calculated after discussion of an application by averaging the overall scores (1-9) given by all voting reviewers on the committee and multiplying by 10. The criterion scores are submitted prior to the meeting by the individual reviewers assigned to an application, and are not discussed specifically at the review meeting or calculated into the overall impact score. Some applications also receive a percentile ranking. For details on the review process, see http://grants.nih.gov/grants/peer_review_process.htm#scoring.

MEETING ROSTER
National Center for Advancing Translational Sciences Special Emphasis Panel

NATIONAL CENTER FOR ADVANCING TRANSLATIONAL SCIENCES
Collaborative Innovation Award (U01 and R21) Review Meeting
ZTR1 CI-4 (01)
03/08/2017 - 03/09/2017

Notice of NIH Policy to All Applicants: Meeting rosters are provided for information purposes only. Applicant investigators and institutional officials must not communicate directly with study section members about an application before or after the review. Failure to observe this policy will create a serious breach of integrity in the peer review process, and may lead to actions outlined in NOT-OD-14-073 at https://grants.nih.gov/grants/guide/notice-files/NOT-OD-14-073.html and NOT-OD-15-106 at https://grants.nih.gov/grants/guide/notice-files/NOT-OD-15-106.html, including removal of the application from immediate review.

CHAIRPERSON(S)

BARRETT, JAMES E., PHD
PROFESSOR AND CHAIR
DEPARTMENT OF PHARMACOLOGY AND PHYSIOLOGY
DIRECTOR, CLINICAL TRANSLATIONAL
RESEARCH INSTITUTE
DREXEL UNIVERSITY COLLEGE OF MEDICINE
PHILADELPHIA, PA 19102

MEMBERS

CSAKY, KARL G., MD, PHD
T. BOONE PICKENS DIRECTOR
CLINICAL CENTER OF INNOVATION FOR AMD
MANAGING DIRECTOR AND CHIEF MEDICAL OFFICER
RETINA FOUNDATION OF THE SOUTHWEST
PARTNER, TEXAS RETINA ASSOCIATES
DALLAS, TX 75231

ELKON, KEITH B., MBBCH, MRCP
PROFESSOR OF MEDICINE AND HEAD
DIVISION OF RHEUMATOLOGY
DEPARTMENT OF MEDICINE
UNIVERSITY OF WASHINGTON
SEATTLE, WA 98195

FIGLIN, ROBERT ALAN, MD
STEVEN SPIELBERG FAMILY CHAIR IN HEMATOLOGY
ONCOLOGY; PROFESSOR OF MEDICINE AND BIOMEDICAL
SCIENCES; DIRECTOR, DIVISION OF HEMATOLOGY
ONCOLOGY; DEPUTY DIRECTOR
MEDICINE AND BIOMEDICAL SCIENCES
DIVISION OF HEMATOLOGY ONCOLOGY
SAMUEL OSCHIN COMPREHENSIVE CANCER INSTITUTE
CEDARS-SINAI MEDICAL CENTER
LOS ANGELES, CA 90048

GODDARD, KATRINA A., PHD
SENIOR INVESTIGATOR
CENTER FOR HEALTH RESEARCH
KAISER PERMANENTE NORTHWEST
PORTLAND, OR 97227

HUANG, KUN, PHD
PROFESSOR
DEPARTMENT OF BIOMEDICAL INFORMATICS
THE OHIO STATE UNIVERSITY
COLUMBUS, OH 43210

HUNTER, LAWRENCE E., PHD
PROFESSOR OF PHARMACOLOGY
DIRECTOR OF COMPUTATIONAL BIOLOGY
GRADUATE TRAINING PROGRAM
UNIVERSITY OF COLORADO DENVER SCHOOL OF MEDICINE
DENVER, CO 80262

MARATHE, MADHAV VISHNU, PHD
PROFESSOR OF COMPUTER SCIENCE
DEPARTMENT OF COMPUTER SCIENCE
VIRGINIA BIOINFORMATICS INSTITUTE
VIRGINIA TECH UNIVERSITY
BLACKSBURG, VA 24060

MCLEROY, KENNETH R., PHD
DISTINGUISHED PROFESSOR AND REGENTS PROFESSOR
DEPARTMENT OF HEALTH PROMOTION AND
COMMUNITY HEALTH SCIENCES
TEXAS A & M UNIVERSITY SYSTEM
SCHOOL OF RURAL PUBLIC HEALTH
COLLEGE STATION, TX 77843

MENON, USHA, BSN, PHD, RN, FAAN
ASSOCIATE DEAN FOR RESEARCH AND GLOBAL ADVANCES
COLLEGE OF NURSING
UNIVERSITY OF ARIZONA
TUCSON, AZ 85721

PIETROBON, RICARDO, MD, PHD
CHIEF EXECUTIVE OFFICER
SPOREDATA INC.
DURHAM, NC 27710

SIEGEL, DAWN H., MD
ASSOCIATE PROFESSOR OF DERMATOLOGY
DEPARTMENT OF DERMATOLOGY
MEDICAL COLLEGE OF WISCONSIN
MILWAUKEE, WI 53226

STOHL, WILLIAM, MD, PHD
PROFESSOR OF MEDICINE
DIVISION OF RHEUMATOLOGY
UNIVERSITY OF SOUTHERN CALIFORNIA
LOS ANGELES, CA 90033

WONG, MITCHELL DAVID, MD, PHD
PROFESSOR OF MEDICINE
DEPARTMENT OF GENERAL INTERNAL MEDICINE
UNVERSITY OF CALIFORNIA LOS ANGELES
LOS ANGELES, CA 90024

MAIL REVIEWER(S)

FASANO, ALESSIO, MD
PROFESSOR AND VICE CHAIR
DEPARTMENT OF PEDIATRICS
MUCOSAL IMMUNOLOGY AND
BIOLOGY RESEARCH CENTER
MASSACHUSETTS GENERAL HOSPITAL – HMS
CHARLESTOWN, MA 02129

SCIENTIFIC REVIEW OFFICER

PONCE, M. LOURDES, PHD
SCIENTIFIC REVIEW OFFICER
OFFICE OF SCIENTIFIC REVIEW
NATIONAL CENTER FOR ADVANCING TRANSLATIONAL
SCIENCES (NCATS)
NATIONAL INSTITUTES OF HEALTH
BETHESDA , MD 20892

EXTRAMURAL SUPPORT ASSISTANT

KRANWINKEL, VICTOR
PROGRAM ASSISTANT
OFFICE OF SCIENTIFIC REVIEW
NATIONAL CENTER FOR ADVANCING TRANSLATIONAL
SCIENCES (NCATS)
NATIONAL INSTITUTES OF HEALTH
BETHESDA, MD 20892

PROGRAM REPRESENTATIVE

BROOKS, PJ, PHD
PROGRAM OFFICER
NATIONAL CENTER FOR ADVANCING
TRANSLATIONAL SCIENCES (NCATS)
NATIONAL INSTITUTES OF HEALTH
BETHESDA, MD 20892

Consultants are required to absent themselves from the room during the review of any application if their presence would constitute or appear to constitute a conflict of interest.
